# Calreticulin is a secreted BMP antagonist, expressed in Hensen's node during neural induction

**DOI:** 10.1016/j.ydbio.2016.12.001

**Published:** 2017-01-15

**Authors:** Irene De Almeida, Nidia M.M. Oliveira, Rebecca A. Randall, Caroline S. Hill, John M. McCoy, Claudio D. Stern

**Affiliations:** a Department of Cell & Developmental Biology, University College London, Gower Street, London WC1E 6BT, UK; b Genetics Institute, Boston, MA, USA; c Francis Crick Institute, Midland Road, London NW1 1AT, UK

**Keywords:** Neural plate, Gastrulation, Neurulation, Chick embryo, Phospho-Smad, Calnexin, Calcium-binding proteins

## Abstract

Hensen's node is the “organizer” of the avian and mammalian early embryo. It has many functions, including neural induction and patterning of the ectoderm and mesoderm. Some of the signals responsible for these activities are known but these do not explain the full complexity of organizer activity. Here we undertake a functional screen to discover new secreted factors expressed by the node at this time of development. Using a Signal Sequence Trap in yeast, we identify several candidates. Here we focus on Calreticulin. We show that in addition to its known functions in intracellular Calcium regulation and protein folding, Calreticulin is secreted, it can bind to BMP4 and act as a BMP antagonist in vivo and in vitro. Calreticulin is not sufficient to account for all organizer functions but may contribute to the complexity of its activity.

## Highlights

• A functional screen uncovers new secreted factors expressed by chick Hensen's node
• A yeast Signal Sequence Trap identifies candidates including Calreticulin and Calnexin.
• We show thatCalreticulin is secretedand binds to BMP4.
• Calreticulin acts as a BMP antagonist in vivo and in vitro.
• Calreticulin does not account for all organizer functions but may contribute to its complexity.

## 1 Introduction

Neural induction is the process by which signals secreted by the organizer (Hensen's node in amniotes, a structure at the tip of the primitive streak) can instruct cells in the epiblast to change their fate from non-neural (eg. epidermis) to neural plate. In chick, a graft of Hensen's node to the proximal anterolateral extraembryonic region (area opaca) can elicit the formation of a complete, patterned nervous system in less than 24 h (for review see Stern, 2005). Although BMP inhibition is absolutely required for neural induction to take place (De Robertis and Kuroda, 2004, Harland, 2000, Hemmati-Brivanlou and Melton, 1997, Linker and Stern, 2004), it is increasingly clear that other signals are also required (Stern, 2005). Known additional factors include FGFs, IGFs and Wnt inhibition, but even a combination of all of these factors is insufficient to mimic the effect of a node graft to the area opaca in the chick (de Almeida et al., 2008, Linker and Stern, 2004), suggesting that other factors are also involved.

To identify new secreted factors expressed in Hensen's node, we took advantage of a Signal Sequence Trap, a rapid strategy to isolate large numbers of cDNAs encoding putative secreted proteins by genetic selection in yeast. A strain of *Saccharomyces cerevisiae* with a genomic deletion at the SUC2 locus (Klein et al., 1996a) is unable to secrete invertase and is therefore unable to grow on sucrose or raffinose as the sole carbon source. A vector with the SUC2 gene lacking the signal sequence and the start codon is then used to construct a library of cDNAs from the tissue of interest. If the cDNA clone provides the elements required for secretion, the fusion protein is translocated to the secretion pathway, allowing the transformant to grow on sucrose or raffinose as their only source of carbon (Jacobs et al., 1997).

Here we use this functional genetic screen to seek new secreted factors from the chick organizer, Hensen's node. Out of 137 putative secreted factors identified, 16 have appropriate expression patterns in the node. These include Calnexin (CANX) and Calreticulin (CALR), molecules previously well studied in connection with intracellular Calcium regulation and glycoprotein folding in the endoplasmic reticulum (Bedard et al., 2005). Misexpression of Calreticulin, but not Calnexin, at the neural plate border can expand the domain of expression of neural plate markers, similar to the effect of BMP antagonists in the same assay. We further show that Calreticulin can be secreted by cells, that it can inhibit BMP, and that soluble Calreticulin can bind to BMP4.

## 2 Materials and methods

### 2.1 Eggs, embryo manipulations and electroporation

Fertilized hens’ eggs (Brown Bovan Gold; Henry Stewart and Company) were incubated at 38 °C to the desired stages, following the Hamburger and Hamilton system (Hamburger and Hamilton, 1951). Electroporation, whole-mount in situ hybridization and whole-mount immunostaining were performed using standard methods as previously described (Sheng et al., 2003, Stern, 1993, Streit and Stern, 2001, Voiculescu et al., 2008). All DNA solutions for electroporation were used at 1.5 μg/µl. FGF8 (50 μg/ml) and Calreticulin (50 μg/ml) proteins were delivered on heparin beads (Sigma; prepared as described by Streit et al., 2000).

### 2.2 Signal Sequence Trap screen and cloning of Calreticulin

**Fig. 1:**
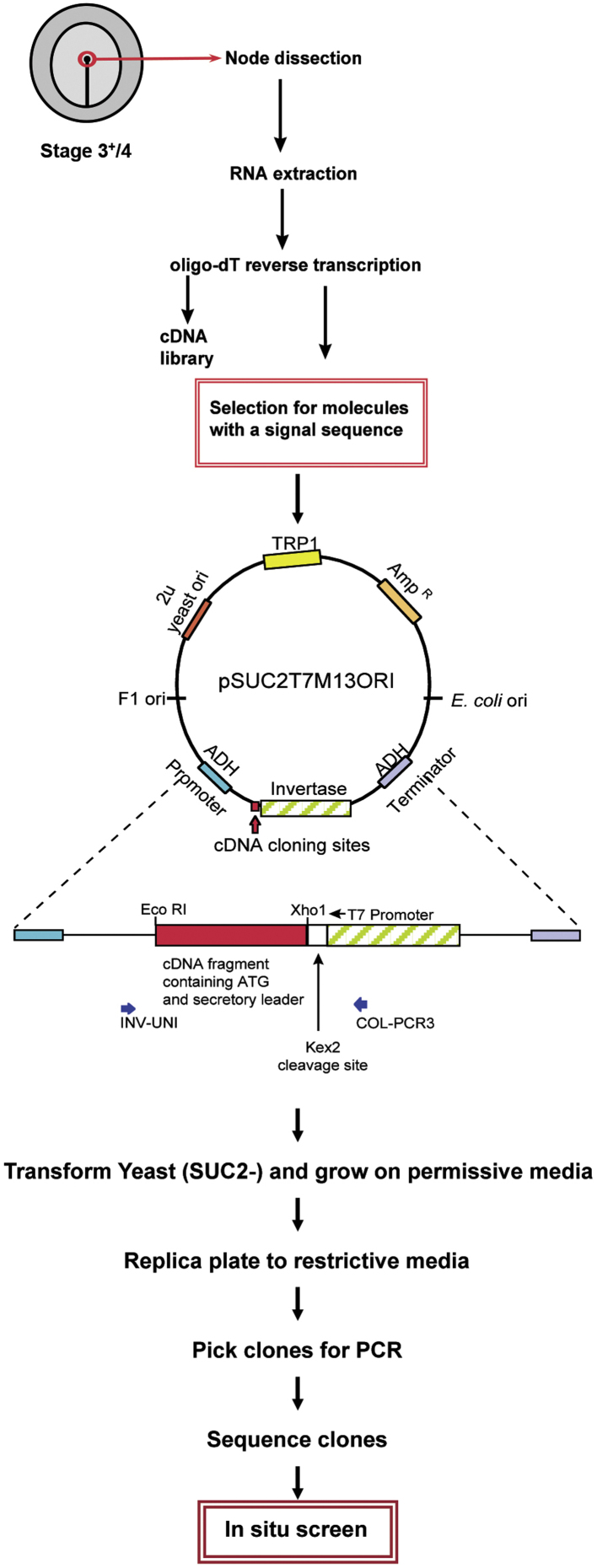
Identification of secreted molecules using the Signal Sequence Trap strategy. Diagram showing the screen methodology: Hensen's nodes were dissected from Stage 3^+^-4 chick embryos; after RNA extraction and reverse transcription the clones were put through the secretion selection and the resulting sequences further screened by in situ hybridization.

A Signal Sequence Trap screen to identify putative secreted factors was performed in yeast as described by Jacobs et al., 1997) (Fig. 1) using a cDNA library constructed by Oligo-dT-primed reverse transcription from mRNA purified from Hensen's nodes of embryos at stage HH3^+^-4. All inserts that passed the selection step (see Fig. 1 and Results) were sequenced and identified initially by BLAST homology searches querying public sequence databases.

Full length Calreticulin was obtained from a stage 2–4 cDNA library as previously described (Streit et al., 2000). The coding regions of chick Calreticulin (CALR), zebrafish Calreticulin (calr) (Rubinstein et al., 2000), human Calnexin (CANX) (kind gift from Marek Michalak (Vassilakos et al., 1998), Xenopus truncated BMP receptor (Suzuki et al., 1994), cSmad6 (a kind gift from P Szendro and G Eichele) (de Almeida et al., 2008, Yamada et al., 1999), cChordin (Streit et al., 1998) and xSmad7 (Casellas and Brivanlou, 1998, de Almeida et al., 2008) were each cloned into pCAβ-IRES-GFP.

The coding region of Calreticulin was also cloned in the pCDNA 3.1/Myc-His (Invitrogen) expression vector using the NotI and BamHI cloning sites. Inserts were generated by PCR using the primers GATCGCGGCCGCATGAGCCGCCTCTGCCTCCCG (adds a NotI restriction site prior to the start codon) and GATCGGATCCTCTTCCTCTCAGCCTCC (removes the stop codon from Calreticulin and adds a BamHI restriction site) and pfuTaq polymerase (Promega) (94 °C, 2 min; 42 °C, 2 min; 72 °C, 2 min; 30 cycles). After digestion of both the PCR fragment and the pCDNA vector with NotI and BamHI, the DNAs were gel purified using a gel extraction kit (Promega) and ligated with T4 ligase (Promega). The resulting plasmid (CALR-Myc) was verified by sequencing.

### 2.3 Cell culture and co-immunoprecipitation

Cell culture and treatments were performed as previously described (Howell et al., 2002) with a few modifications: HEK-293T cells were cultured in Dulbecco's modified Eagle's medium (DMEM) containing 10% fetal bovine serum and transfected using Lipofectamine™ 2000 in combination with Plus Reagent (Invitrogen) according to the manufacturer's instructions. Cells were seeded at 10^5^ cells per well in a 6-well plate. The next day, each well was transfected with 1 µg DNA (either control vector [no insert], dominant-negative BMP receptor (dnBMPr), CANX or CALR, all in pCAβ-[insert]-IRES-GFP). To study BMP inhibition, transfected cells were grown for 72 h and then treated with human BMP4 protein (R&D Systems) at 20 ng/ml for 1 h prior to preparation of whole cell extracts. Cell lyses and Western blots were performed as previously described (Howell et al., 2002).

COS cells were transfected with CALR-Myc, Chordin-Myc (Streit et al., 1998) or control, empty Vector by a similar method. Pellets of transfected COS cells for grafting into embryos were generated from hanging drops as previously described (Streit et al., 1998, Streit and Stern, 1999).

For co-immunoprecipitations (Co-IP), 500 µl of lysates from transfected cells were clarified by centrifugation for 10 min at 13,200 r.p.m. at 4 °C and diluted (1:2) in Co-IP wash buffer (0.025 M Tris pH7.4, 0.15 M NaCl, 0.001 M EDTA, 1% NP‐40 and 5% glycerol). The sample was first immunoprecipitated with 1–2 µg of the protein-specific antibody (either mouse anti-BMP4 IgG, Enzo Life Sciences or rabbit anti-Calreticulin, Cell Signalling Technology or control mouse or rabbit Ig [mouse IgG from Santa Cruz, Rb IgG from BD Pharmigen]) overnight at 4 °C on a rocking platform. As additional negative controls, we used cell lysates of non-transfected HEK-293T cells. Recombinant BMP4 (Biotechne) and Calreticulin (Abcam) were used as positive controls, diluted in Co-IP wash buffer (up to 1 µg/ml). The precipitated supernatants (1 ml) were complexed with 20 µl pre-washed Sepharose L (Santa Cruz) and incubated for 3 h at 4 °C on a rocking platform. After centrifugation and removal of most of the supernatant, the beads were eluted from the Sepharose by addition of 50 µl of 2×-concentrated sample buffer (stock: 100 mM Tris pH6.8, 4% SDS, 0.2% Bromophenol Blue, 20% glycerol, 20 mM β-mercaptoethanol) for 1 h at 50 °C. The Sepharose was centrifuged for 1 min at 13,000 r.p.m. and the supernatants harvested and stored at −20°C until ready for Western Blotting. Antibodies against PhosphoSmad1/5/8, Smad1, Calreticulin, BMP4 (all from Cell Signaling Technology), GFP (Life Technologies) and Goat anti-rabbit HRP (Santa Cruz) were used at 1:1000 for Western blots and 1:2000 for immunohistochemistry. A monoclonal anti-Myc antibody (9E10; Developmental Studies Hybridoma Bank) was used as a 1:1 dilution of supernatant from the hybridoma cells.

## 3 Results

### 3.1 A Signal Sequence Trap screen to identify putative secreted molecules in Hensen's node

To identify cDNAs encoding candidate secreted molecules in Hensen's node at the stage when neural induction takes place, a cDNA library from stage 3^+^-4 chick nodes was constructed and introduced into the pSUC2T7M13ORI vector for yeast transfection as described by Jacobs et al., 1997) (Fig. 1). After growth in restrictive medium (to enrich for putative secreted proteins), 442 inserts were selected. These were sequenced and identified initially by BLAST. Of these, 137 clones (31% of the inserts) were selected for further analysis (Supplementary Table 1); the remainder encoded either 18S RNA sequences or mitochondrial sequences.

The 137 sequences were further analysed for the presence of a signal sequence using the SignalP 3.0 algorithm (http://www.cbs.dtu.dk/services/SignalP/) (Bendtsen et al., 2004, Nielsen et al., 1997a, Nielsen et al., 1997b). One hundred and three sequences (75%) either encode known secreted proteins or are predicted to be secreted (Supplementary Table 1, column headed SP). The proportion of putative secreted candidates is similar to that obtained by others using comparable screens (Kaiser et al., 1987).

As a secondary screen for factors that could be involved in neural induction, we conducted whole-mount in situ hybridization on embryos from before primitive streak formation to about stage 5 (Hamburger and Hamilton, 1951), to determine whether the mRNA is transcribed in the node at stages 3^+^-4, when the inducing ability of the node is strongest (Storey et al., 1992). Sixteen of the transcripts are enriched in Hensen's node, making them possible candidate molecules to study in the context of neural induction (Supplementary Fig. 1).

### 3.2 Calreticulin is expressed in Hensen's node

Of the 16 clones with expression in Hensen's node, two (nhbr307 and nhbw87; Supplementary Fig. 1) encode Calreticulin (CALR) and Calnexin (CANX) respectively; both are well-studied proteins involved in protein quality control and Calcium regulation (Chevet et al., 2009, Fliegel et al., 1989a, Fliegel et al., 1989b, MacLennan et al., 1974, Michalak et al., 2009, Ostwald and MacLennan, 1974, Smith et al., 1989, Wada et al., 1991).

**Fig. 2:**
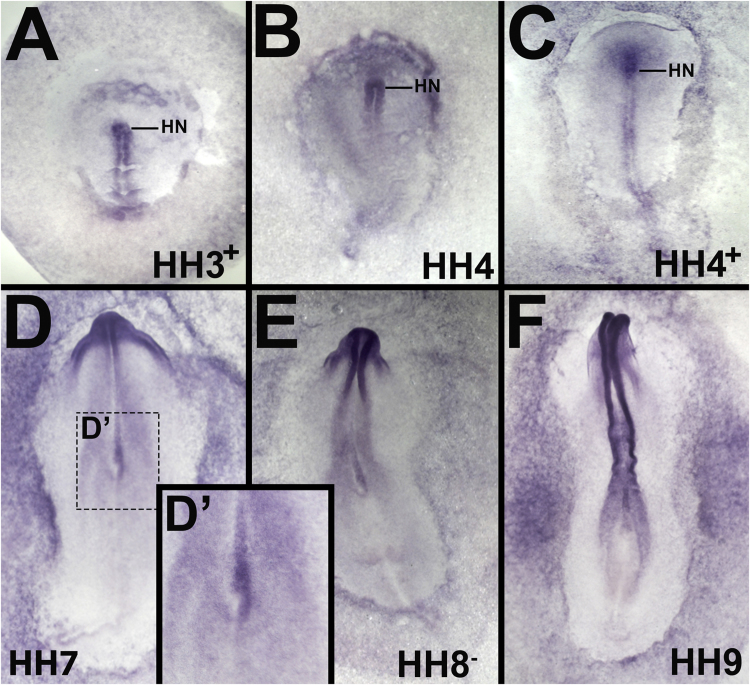
Calreticulin is expressed in Hensen's node and neural plate in the chick embryo. Calreticulin transcripts are detected at stage 3-3^+^(A) with a stronger signal in the anterior part of the primitive streak. At stages 4-4^+^, expression becomes concentrated in Hensen's node, with lower levels in the anterior epiblast (B, C). D. From stage 7, expression in Hensen's node is asymmetric, concentrated on the right side (D, D‘, E). After this stage, expression is concentrated in the neural plate (E, F). HN, Hensen's node.

To assess the expression of Calreticulin in more detail at early stages of chick development, whole mount in situ hybridization was performed using a full-length CALR clone. Weak expression is first detected throughout the area pellucida epiblast before primitive streak formation (Supplementary Fig. 1). At stages 3^+^*-*4^+^, expression becomes concentrated in the anterior primitive streak including Hensen's node (Fig. 2A-C). At stage 7–8, expression in the node is strongly asymmetric, concentrated on the right side (Fig. 2D, D′, E). From stage 8, expression is also seen in the neural plate (Fig. 2E-F) and more weakly in extraembryonic and lateral mesoderm. From stage 10, expression appears to become ubiquitous (not shown).

Since Calnexin (which was also identified in the screen as a possible secreted molecule in Hensen's node) shares a number of properties with Calreticulin (Wada et al., 1991), we studied its expression. Calnexin mRNA levels are weak at early stages of development although transcripts are slightly concentrated in the node around stage 4 (Supplementary Fig. 2A-C). Thereafter expression becomes more widespread and becomes concentrated in the neural plate and extraembryonic and lateral mesoderm (Supplementary Fig. 2 D-F).

### 3.3 Calreticulin, but not Calnexin, expands the neural plate

**Fig. 3:**
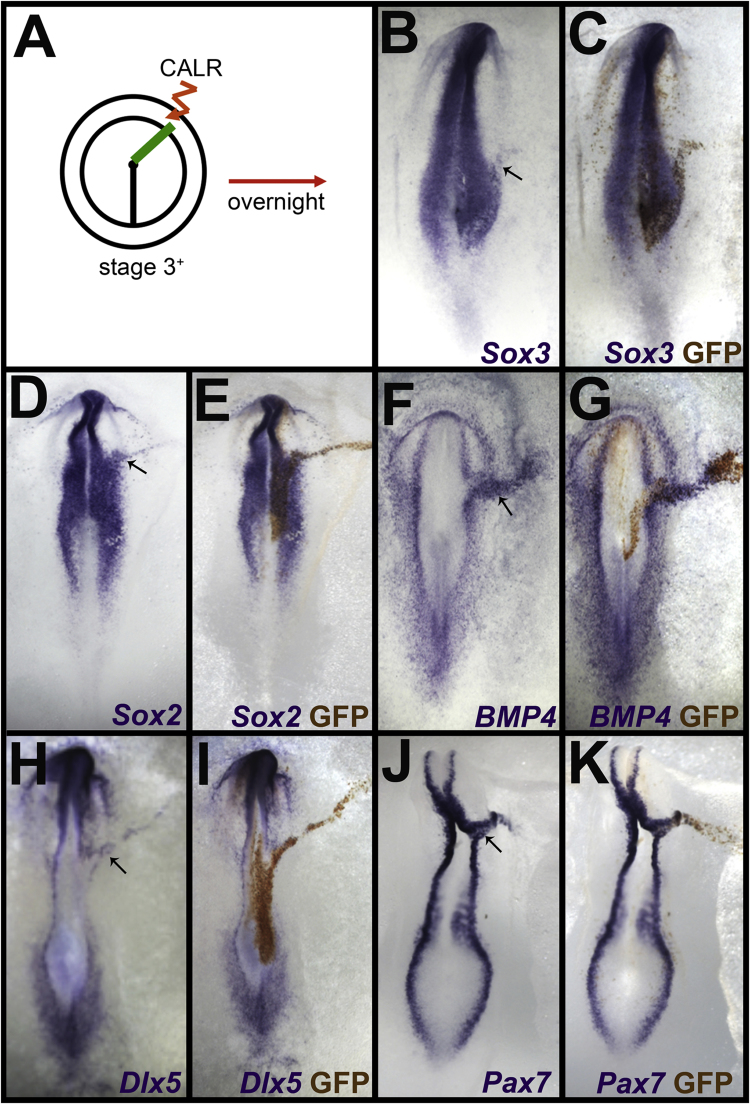
Calreticulin misexpression extends the neural plate. A. Calreticulin (CALR) was electroporated into the epiblast of stage 3^+^ chick embryos in a line extending from the node to the area opaca. B-E. Misexpression causes expansion of the neural plate markers Sox3 (B, C) and Sox2 (D, E). F-K. Along with this, the neural plate border markers BMP4 (F, G), Dlx5 (H, I) and Pax7 (J, K) are displaced laterally. Note that neither effect extends to the area opaca. In B-K, purple shows the mRNA (in situ hybridization) and brown is anti-GFP to reveal the electroporated cells. The arrows in B, D, F, H and J point to the lateral expansion of the marker.

Several molecules with a role in neural induction, such as BMP antagonists, can expand the neural plate (and laterally displace its border) into the non-neural territory when misexpressed as a line, extending outwards from the prospective neural plate (de Almeida et al., 2008, Delaune et al., 2005, Fisher and Halpern, 1999, Furthauer et al., 1999, Kuroda et al., 2004, Linker et al., 2009, Linker and Stern, 2004, Papanayotou et al., 2013, Papanayotou et al., 2008, Piccolo et al., 1996, Streit et al., 1998, Streit and Stern, 1999). To determine whether Calreticulin can do this, a bicistronic vector encoding Calreticulin and GFP (pCAβ-*CALR*-IRES-GFP) was electroporated into the epiblast of stage 3^+^ chick embryos in a line extending from within the prospective neural plate to the area opaca (Fig. 3A). This causes expansion of neural plate markers like Sox3 (7/15 embryos, 47%; Fig. 3B-C) and Sox2 (9/20, 45%; Fig. 3D-E) into the non-neural ectoderm. Along with the neural plate, the neural plate border is also displaced laterally as assessed with msx1 (5/12 cases, 42%; not shown), BMP4 (5/11, 45%; Fig. 3F-G), Pax7 (6/10, 60%; Fig. 3H-I) and Dlx5 (6/12, 50%; Fig. 3J-K). However, Slug is downregulated, especially posteriorly (3/7 - 43%; not shown). Notably, although expansion of the neural plate and lateral shift of its border was very extensive, the effect never extended to the area opaca. In control embryos electroporated with the control vector control (pCAβ-IRES-GFP), no change was seen in expression of Sox3 (0/4), Sox2 (0/8), BMP4 (0/5), Dlx5 (0/8), Pax7 (0/8), Msx1 (0/7) or Slug (0/4).

We also tested whether Calnexin can expand the neural plate. Electroporation of pCAβ-CANX-IRES-GFP as a line into the epiblast of stage 3^+^ chick embryos (as described above, has no effect on Sox3 (0/12; not shown) or Sox2 (0/14; Fig. 2G, H), which showed no difference to control electroporated embryos (0/8 for Sox3, not shown; 0/8 for Sox2, Supplementary Fig. 2I, J).

### 3.4 Calreticulin is insufficient for neural induction in the area opaca even in combination with FGF and BMP antagonists

**Fig. 4:**
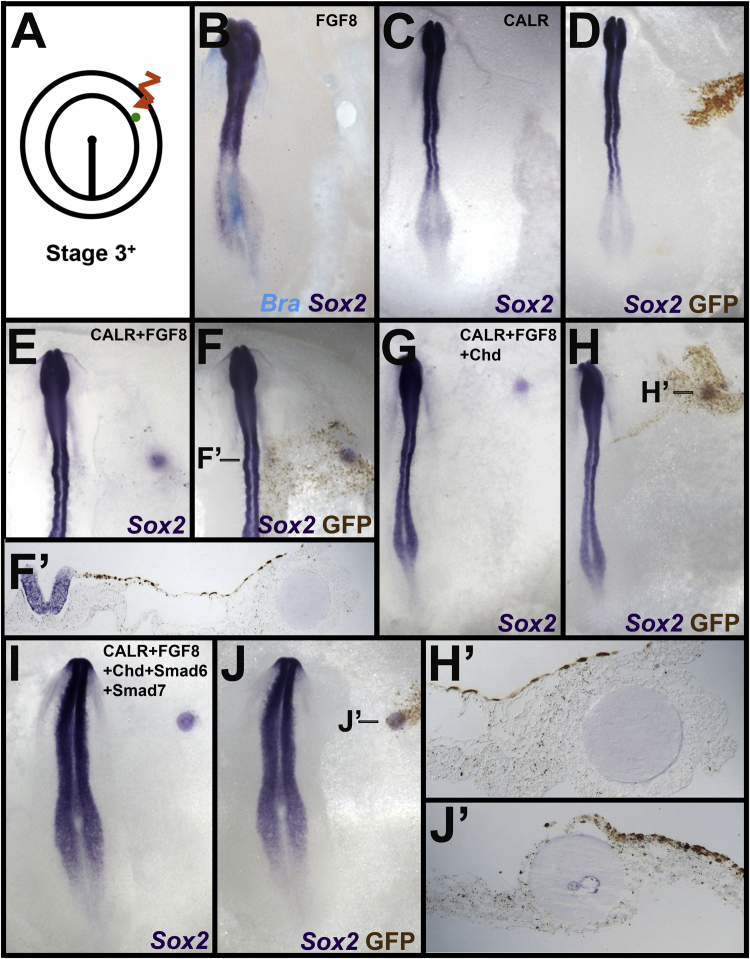
Calreticulin, either alone or together with FGF and BMP antagonists, cannot induce neural markers in the area opaca. A. Experiments (electroporation and/or a graft of protein-loaded beads) were performed in the competent extraembryonic epiblast of the area opaca at stage 3^+^. B. FGF8 alone does not induce the neural marker Sox2 or the mesodermal marker Brachyury (Bra) in the area opaca. Calreticulin electroporation does not induce Sox2 in the area opaca either by itself (C, D), or when combined with FGF8 (E, F) or with both FGF plus the BMP antagonists Chd (G, H) and/or Smad6 and Smad7 (I, J). Note that the beads themselves lightly stain during the in situ process (E, G, I); histological sections show the absence of expression in the epiblast above the beads (F′, H′ and J′).

When a graft of Hensen's node is placed into the proximal anterior area opaca, it can induce a full range of neural and border markers within about 12 h (Dias and Schoenwolf, 1990, Gallera, 1971, Linker et al., 2009, Linker and Stern, 2004, Pinho et al., 2011, Storey et al., 1992, Streit et al., 1998, Streit et al., 1997, Streit and Stern, 1999). To test whether Calreticulin misexpression can induce the competent epiblast of the area opaca to acquire expression of neural markers, pCAβ-CALR-IRES-GFP was electroporated into a discrete domain within the inner third of area opaca at stage 3^+^, just anterior to the level of Hensen's node (Fig. 4A). After incubation for 15–20 h, no expression of Sox3 (0/5, not shown) or Sox2 (0/8; Fig. 4C-D) was observed in the electroporated region. Control electroporations with pCAβ-IRES-GFP also had no effect on Sox3 (0/5) or on Sox2 (0/4) expression (not shown).

FGF signalling is required for the initial steps of neural induction as well as for cells to respond to BMP antagonists (de Almeida et al., 2008, Delaune et al., 2005, Lamb and Harland, 1995, Launay et al., 1996, Linker et al., 2009, Linker and Stern, 2004, Papanayotou et al., 2008, Sasai et al., 1996, Sheng et al., 2003, Streit et al., 2000). The failure of Calreticulin alone to induce neural markers in the area opaca (see above) raises the possibility that additional factors are required, such as FGF and/or BMP antagonism. A graft of a heparin bead soaked in FGF by itself does not induce either Sox2 or the mesodermal marker Brachyury (0/5 of each marker; Fig. 4B-C). A combination of a FGF bead with electroporation of Calreticulin still fails to induce Sox2 in the area opaca (0/10; Fig. 4E, F, F‘). Calreticulin was also unable to induce Sox2 in the presence of both Chordin and FGF8 (0/10; Fig. 4G, H, H‘). Even a combination of Calreticulin with FGF8 and the BMP antagonists Chordin (Streit et al., 1998), Smad6 (Imamura et al., 1997) and Smad7 (Casellas and Brivanlou, 1998) is unable to induce Sox2 (0/14; Fig. 4I, J, J‘) in the area opaca epiblast. These results show that Calreticulin is unable to induce Sox2 in area opaca epiblast even in combination with FGF and BMP inhibitors.

### 3.5 Calreticulin can be secreted by cells

**Fig. 5:**
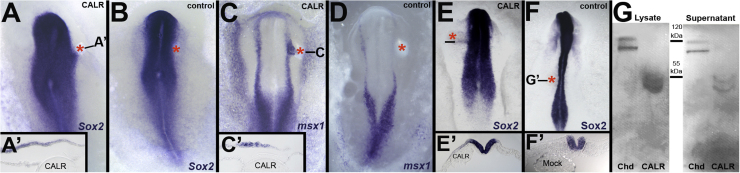
Calreticulin is secreted and soluble protein can expand the neural plate. A-D. Soluble Calreticulin can expand the neural plate. Heparin beads soaked in Calreticulin placed at the boundary of the prospective neural epiblast expand Sox2 expression laterally (A, A′), unlike control beads (B). A similar effect is revealed with the border marker msx1 (C, C′) and this is also not seen in controls (D). E-G. Calreticulin can be secreted from cells. E-F. Calreticulin-transfected COS cells also cause expansion of the neural plate (E, E′), unlike control COS cells (F, F′). Pellets of cells and heparin beads are highlighted with a red asterisk. G. COS cells were transfected with Myc-tagged Calreticulin and cultured, and the cell lysate and supernatant were analysed for Calreticulin in Western blots probed for the Myc tag. A 55 kDa band corresponding to Calreticulin is recovered from both the lysate and supernatant. Myc-tagged Chordin (100 and 120 kDa bands) was used as a positive control.

Although Calreticulin is generally viewed as an endoplasmic reticulum-associated protein (Michalak et al., 2009), its isolation in the yeast Signal Sequence Trap screen raises the possibility that Calreticulin might be a secreted protein, as also suggested by previous authors (Čiplys et al., 2015, Gold et al., 2010). To test whether expansion of the neural plate can be elicited by soluble, extracellular Calreticulin, we tested the effect of heparin-beads loaded with recombinant Calreticulin protein, grafted close to the prospective neural plate border. This mimics the effect of misexpression by electroporation: the domain of Sox2 expression is expanded (4/4; Fig. 5A, A′) and the border marker msx1 (4/5; Fig. 5C, C′) is shifted laterally. Control grafted beads did not alter the expression of either Sox2 (0/5; Fig. 5B) or msx1 (0/4; Fig. 5D).

Next, to test whether Calreticulin can be secreted by cells, we first examined whether a graft of a pellet of COS cells that had been transfected with CALR could mimic this effect in embryos. In 3/11 embryos, the pellet caused expansion of Sox2 expression in epiblast adjacent to the grafted cells (Fig. 5E). Control embryos were unaffected (0/6; Fig. 5F). This weak effect could be due to low transfection efficiency of the COS cells and/or to low levels of secretion by these cells and/or to loss of the expression construct during the culture period required to make the hanging drop pellets. As a more direct test for secretion, we plated freshly-transfected COS cells and collected the supernatant and a lysate of the cells themselves and tested for the presence of CALR-Myc by Western blotting. As a positive control, we used Chordin-Myc. Both Chordin-Myc and CALR-Myc could be detected in the supernatant of transfected cells (Fig. 5G), confirming that Calreticulin protein can be secreted from cells.

### 3.6 Calreticulin acts as a BMP antagonist

**Fig. 6:**
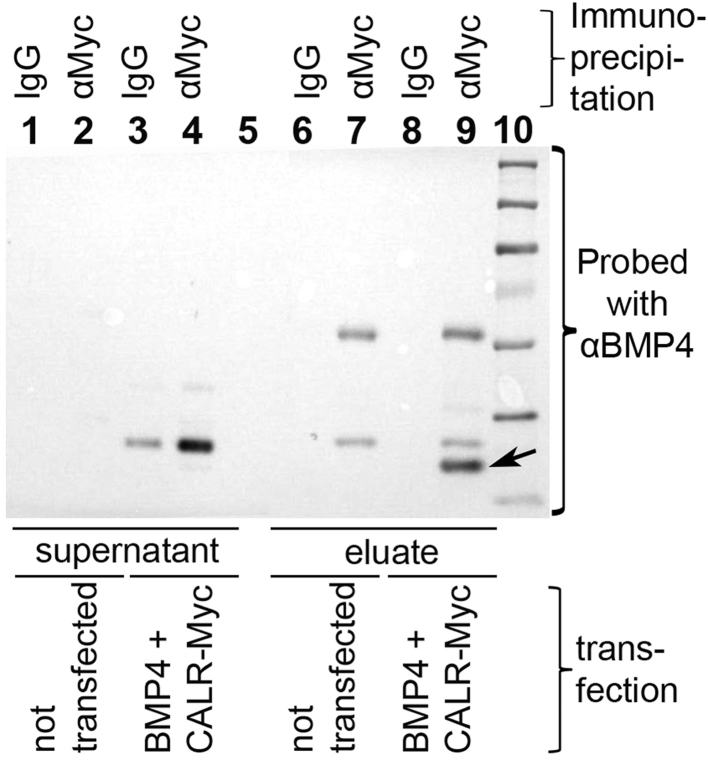
Calreticulin physically interacts with BMP4. HEK-293T cells were co-transfected with expression constructs encoding BMP4 and Myc-tagged Calreticulin. After immunoprecipitation with anti-Myc antibody (lanes 2, 4, 7, 9) or mouse IgG as control (lanes 1, 3, 6, 8), the supernatant not bound to the Myc-beads (lanes 1–4) and the eluate from the Myc-beads (lanes 6–9) were analysed by Western blotting, probed with an antibody to BMP4. A unique band of 23 kDa is precipitated from the co-transfected cells, corresponding to BMP4 (arrow). Lane 10 contains molecular weight markers.

The above results suggest that Calreticulin can be secreted from cells, and that misexpression (either by electroporation or applied as an extracellular signal) can expand the neural plate and displace its border laterally. These effects are similar to those of BMP antagonists like Chordin and Noggin (Lamb et al., 1993, Sasai et al., 1994, Streit et al., 1998, Streit and Stern, 1999), raising the possibility that soluble Calreticulin could act as a BMP antagonist, perhaps by binding to this protein and sequestering it away from the receptors. To test this, we explored whether Calreticulin protein can bind directly to BMP4 by co-immunoprecipitation (Fig. 6). HEK-293T cells were transfected with CALR-Myc and BMP4, the medium immunoprecipitated with anti-Myc and Western blots of protein eluted from the Sepharose beads probed with anti-BMP4 antibody. A 23 kDa band corresponding to BMP4 is precipitated (lane 9, arrow). This band is absent from controls: supernatant from untransfected cells (lanes 1–2) or from transfected cells (lanes 3–4), Sepharose-bound eluate from untransfected cells (lanes 6–7), precipitated with control IgG antibody (lanes 1, 3, 6) or with anti-Myc antibody (lanes 2, 4, 7). It is also absent from the eluate of transfected cells precipitated with control antibody (lane 8). This experiment shows that Calreticulin secreted from cells can bind to BMP4.

**Fig. 7:**
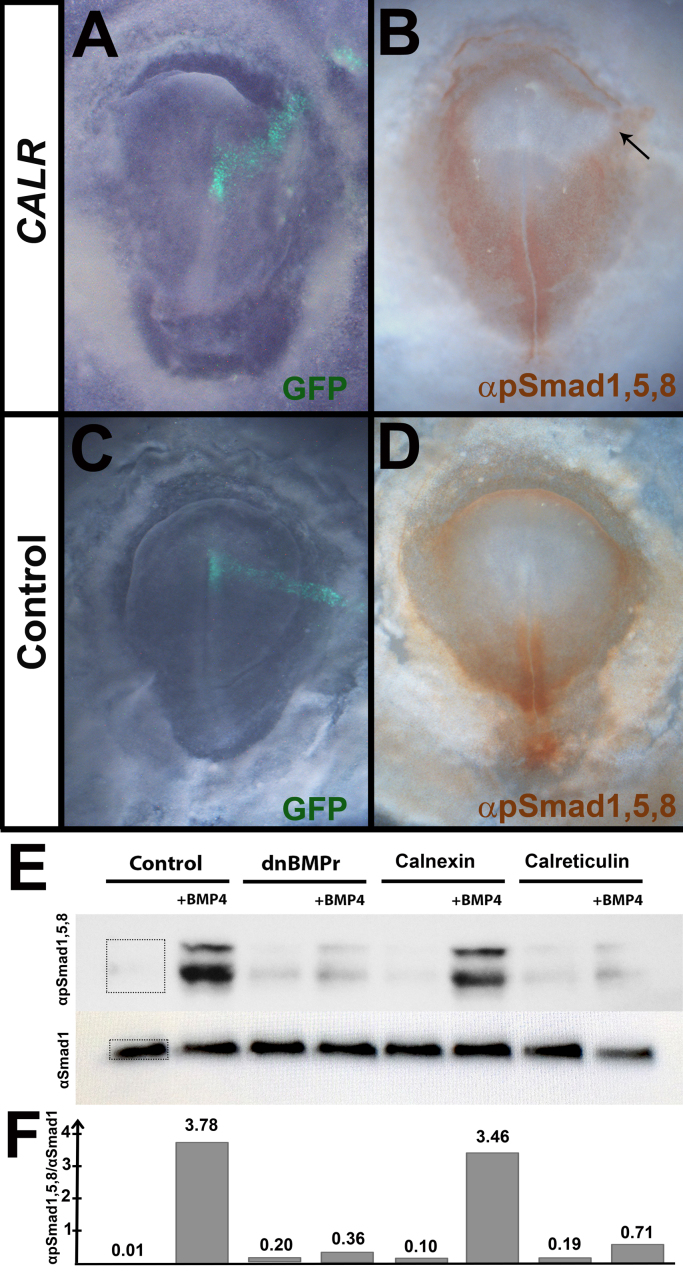
Calreticulin inhibits the BMP pathway, both in vivo and in vitro. A-D. When Calreticulin is electroporated as a line extending from the midline to the area opaca and the embryos cultured for 6–8 h, BMP signalling is inhibited in the electroporated area, as marked by the loss of anti-phospho-Smad1/5/8 staining in the electroporated region (corresponding to expansion of the neural plate; arrow in B) (A, B). This is not seen in control embryos, electroporated with empty vector (C, D). E-F. Calreticulin can lower the levels of phospho-Smad1/5/8 in vitro. HEK-293T cells transfected with either dnBMPr (as positive control) or Calreticulin, when stimulated with 20 ng/ml BMP4 for 1 h, show repression of the BMP signalling, as marked by the lower levels of phosphorylated Smad 1/5/8. Cells transfected with an empty vector or Calnexin do not downregulate the BMP pathway. The same blot was stripped and re-probed with an antibody against Smad1 (recognising both phosphorylated and non-phosphorylated forms) as a loading control. E shows the blots, and F shows the ratio between αpSmad1,5,8 and αSmad1 to quantify relative phosphorylation signal in each sample. This was calculated by dividing the optical density within the box shown in the αpSmad1,5,8 of the first lane over the corresponding αSmad1 (boxes shown for lane 1). Identical boxes were used for each lane. The number above each bin is the ratio.

To test more directly whether Calreticulin can inhibit BMP activity in vivo, we repeated the Calreticulin misexpression experiment (a line extending laterally from the neural plate) and cultured the embryos for 6–8 h. The embryos were then fixed and stained with an antibody against phospho-Smad1/5/8 (Faure et al., 2002), since BMP signalling acts by phosphorylating these intracellular proteins (von Bubnoff and Cho, 2001). Electroporation (green cells in Fig. 7A) caused lateral expansion of the region devoid of phospho-Smad1/5/8 (Fig. 7B). Control embryos (electroporated with pCAβ-IRES-GFP) are unaffected (Fig. 7 C-D).

We also tested the ability of Calreticulin to modulate BMP4 activity in vitro. HEK-293T cells were transfected with various constructs: empty vector (negative control), dnBMP-receptor (dnBMPr; positive control), Calnexin and Calreticulin. After 72 h the cells were stimulated with 20 ng/ml BMP4 protein for 1 h applied to the medium, harvested and the lysates analysed by Western blotting for PhosphoSmad1/5/8. Both dnBMPr and Calreticulin repressed the BMP response, as revealed by the reduced levels of phospho-Smad after BMP stimulation. In contrast, Calnexin does not inhibit BMP activity (Fig. 7E, F).

In conclusion, our results show that Calreticulin is expressed in Hensen's node during the stages of neural induction, that it can be secreted by cells, and that it can expand the neural plate when misexpressed close to its border. This latter effect resembles that of BMP antagonists and we show that Calreticulin can indeed bind to BMP4 and inhibit BMP activity in vivo and in vitro. Together these results implicate Calreticulin from the node as an additional BMP antagonist that may contribute to the neural induction process.

## 4 Discussion

### 4.1 The Signal Sequence Trap as a method to identify secreted molecules

Most eukaryotic secreted proteins (or those that are membrane bound) contain amino-terminal or internal signal peptides that direct their sorting to the endoplasmic reticulum (ER). From the ER, proteins are transported to the extracellular space or the plasma membrane through the ER-Golgi secretory pathway (Lee et al., 2004, Osborne et al., 2005). Here we took advantage of a Signal Sequence Trap method in yeast (Jacobs et al., 1997, Klein et al., 1996b) as a rapid and relatively simple method for isolating large numbers of cDNAs that might encode for secreted proteins. It has been used successfully to identify novel signalling components during vertebrate development including Derrière (Sun et al., 1999), Frizzled/Sizzled (Bradley et al., 2000) and CRISP (Smith et al., 2001) proteins.

Kaiser et al. (1987) have shown that about 20% of a large number of sequences can function to cause secretion in yeast if an initiating methionine is provided and that many random sequences functionally replace the secretion signal sequence of yeast invertase. This can lead to the isolation of many false positives in this screen. In our case, the majority of the sequences isolated (305/442) encoded either ribosomal or mitochondrial sequences. Of the remaining 137, 103 (75%) were either known to be secreted proteins or contained a sequence predicted to direct secretion. We were surprised that none of the well-studied secreted proteins expressed in Hensen's node (such as FGF8, Chordin, ADMP or Sonic hedgehog) were identified in the screen. It is possible that some cDNAs did not fuse appropriately in frame with the invertase. It is also possible that the yeast translational machinery is unable to generate proteins from some heterologous mRNA sequences. Another possibility is that some vertebrate signal sequences are not active in the yeast secretion machinery, which has been shown for some human proteins (Born et al., 1987).

Using this screening method, we identified Calreticulin. We show that it is expressed in Hensen's node at the time of neural induction. When the chick Calreticulin clone is misexpressed in COS or 293T cells, the protein can be retrieved from the supernatant and this supernatant is active as a BMP antagonist. Moreover, when these transfected cells are transplanted into an embryo, they can expand the neural plate and inhibit BMP signalling in vivo. This effect can also be demonstrated using purified Calreticulin protein applied locally to the embryo. These results show that Calreticulin can be secreted from cells. Indeed, in addition to its better known functions in the ER (Gao et al., 2002, Ireland et al., 2008, Michalak et al., 2009, Molinari et al., 2004, Tannous et al., 2015), Calreticulin plays other very important roles outside the ER, both in physiological and in stress-related conditions and disease (Gold et al., 2010). For example, secreted and membrane-associated forms of Calreticulin are important in the host immune response to cancer with respect to activation of T cells, peptide loading with tumour antigens and in the phagocytosis of tumour cells by dendritic cells, which has been used to design chemotherapeutic approaches (Chaput et al., 2007, Feng et al., 2015, Gardai et al., 2005, Obeid et al., 2007, Wemeau et al., 2010). Extracellular Calreticulin also plays an important role in cutaneous wound healing (Greives et al., 2012, Nanney et al., 2008). These observations add significant strength to our proposal that Calreticulin can be secreted, and that it may have non-cell-autonomous functions during early development.

### 4.2 Functions of Calreticulin in development

Calreticulin appears to fulfil many cellular functions, both within and outside the ER. In the ER lumen it performs two major functions: chaperoning and regulation of Ca^2+^ homeostasis (Mery et al., 1996, Wada et al., 1994). Calreticulin is a highly versatile lectin-like chaperone (Hebert et al., 1995, Peterson et al., 1995, Spiro et al., 1996) and it participates in the synthesis of a variety of molecules, including ion channels, surface receptors, integrin and transporters. The protein also affects intracellular Ca^2+^ homeostasis by modulation of ER Ca^2+^ storage and transport. Upregulation of Calreticulin leads to numerous effects in different experimental models, including increased Ca^2+^ storage capacity of the ER (Mery et al., 1996), modulation of cell adhesion (Leung-Hagesteijn et al., 1994), modulation of store-operated Ca^2+^ influx (Mery et al., 2005, Mery et al., 1996), increased propensity to apoptosis (Knee et al., 2003), modulation of steroid sensitive gene expression (Burns et al., 1994) and modulation of the function of another ER calcium pump, SERCA2 (John et al., 1998). In cell culture, downregulation of Calreticulin causes changes in cell adhesion (Coppolino et al., 1997, Rauch et al., 2000), increased resistance to apoptosis (Nakamura et al., 2000), abnormal accumulation of misfolded proteins (Knee et al., 2003), modulation of Ca^2+^-dependent gene transcription (Mesaeli et al., 1999) and inhibition of agonist-dependent Ca^2+^ release from ER stores (Mesaeli et al., 1999).

However almost nothing is known about the functions of Calreticulin during development. Homozygous Calreticulin mutant mice die at E14.5 (Mesaeli et al., 1999) with impaired cardiac development and problems in Ca^2+^-dependent transcriptional pathways (Guo et al., 2002, Mesaeli et al., 2001, Mesaeli et al., 1999), and also display brain and body wall defects (Rauch et al., 2000). A zebrafish Calreticulin homologue was first isolated in a screen for genes whose expression is dependent on Cyclops signalling (a Nodal homologue); it is expressed in the embryonic shield (the zebrafish organizer) but no function was assigned to it (Rubinstein et al., 2000).

Our results reveal that Calreticulin is expressed in Hensen's node, that it can be secreted from cells and that soluble Calreticulin can act as a BMP antagonist both in vivo and in vitro. Since Calreticulin null mutant mice do not fail to form a nervous system, it is unlikely that Calreticulin is the most important single BMP inhibitor expressed in the organizer at the time of neural induction. However at this stage the node co-expresses Chordin (Streit et al., 1998), making it likely that the roles of these two inhibitors partly overlap or are synergistic. Since Chordin mutants have severe defects but do not fail to induce a nervous system (Bachiller et al., 2000), it may be interesting to explore the phenotype of Chordin-Calreticulin double-mutants. Nevertheless, our results point to a previously undescribed function of Calreticulin, which may be more widespread than just during development.
