# Supplementary material for: Calreticulin is a secreted BMP antagonist, expressed in Hensen's node during neural induction
Source: Dev Biol. 2017 Jan 15;421(2):161–70. doi: 10.1016/j.ydbio.2016.12.001 (PMC5231319; doi:10.1016/j.ydbio.2016.12.001)
Supplement: Supplementary file 1 — Supplementary Information [file mmc1.pdf]

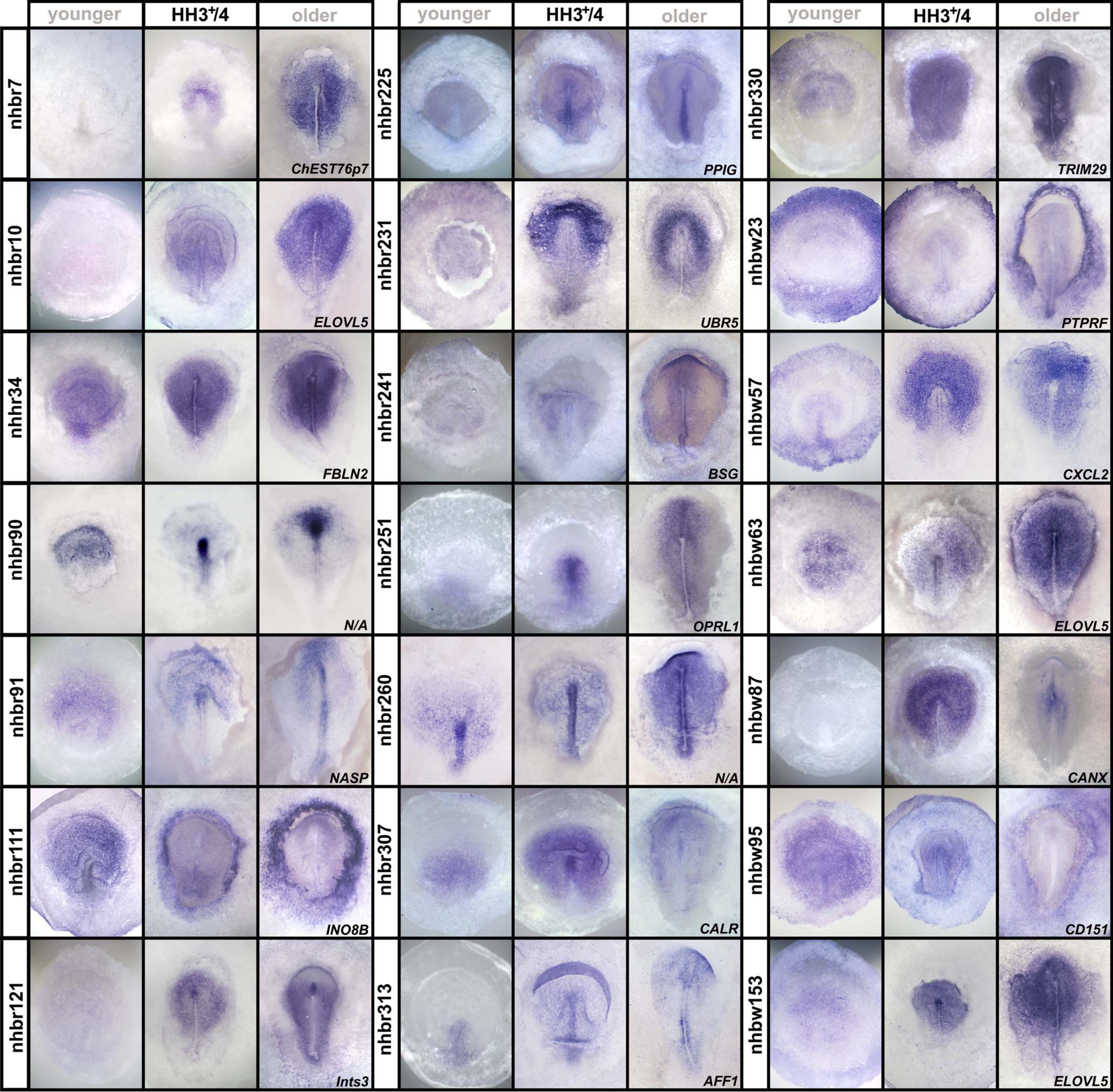
younger
HH3+/4
older
younger
HH3+/4
older
younger
HH3+/4
older
nhbr7
ChEST76p7
nhbr225
PPIG
nhbr330
TRIM29
nhbr10
ELOVL5
nhbr231
UBR5
nhbw23
PTPRF
nhhr34
FBLN2
nhbr241
BSG
nhbw57
CXCL2
nhbr90
N/A
nhbr251
OPRL1
nhbw63
ELOVL5
nhbr91
NASP
nhbr260
N/A
nhbw87
CANX
nhbr111
INO8B
nhbr307
CALR
nhbw95
CD151
nhbr121
Ints3
nhbr313
AFF1
nhbw153
ELOVL5

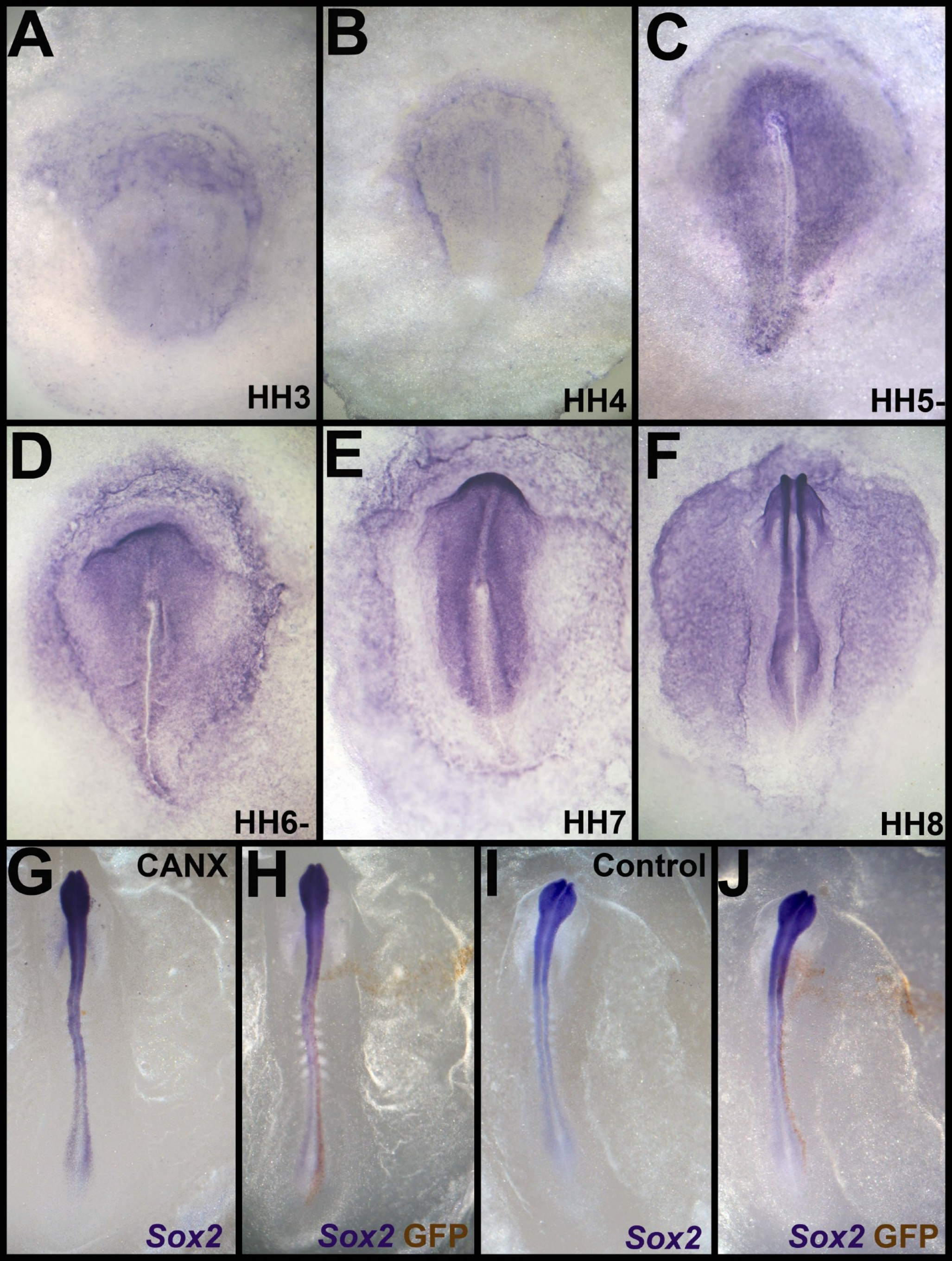
A
HH3
B
HH4
C
HH5-
D
HH6-
E
HH7
F
HH8
G
CANX
Sox2
H
Sox2 GFP
I
Control
Sox2
J
Sox2 GFP
